# How does the ultrasonic assist CO_2_ immiscible flooding?

**DOI:** 10.1016/j.ultsonch.2025.107260

**Published:** 2025-02-07

**Authors:** Hengli Wang, Leng Tian, Yanzhong Zhen, Yating Li, Yi Gao, Gaorun Zhong, Kaiqiang Zhang

**Affiliations:** a School of Petroleum Engineering and Environmental Engineering, Yan’An University, Yan’An 716000 PR China; b Department of Petroleum Engineering, China University of Petroleum (Beijing), Beijing 102249 PR China; c Yanchang Oilfield Co LTD 716000 PR Chian; d Institute of Energy, Peking University, Beijing 100871 PR China; e Institute of Carbon Neutrality, Peking University, Beijing, PR China; f Ordos Research Institute of Energy, Peking University, Ordos, PR China

**Keywords:** CO_2_ immiscible flooding, Ultrasonic assisted, Visible porous media, EOR mechanism

## Abstract

CO_2_ flooding is considered one of the most important methods for reducing CO_2_ emissions and increasing oil production from reservoirs. However, the challenges of low recovery rates and poor economic benefits in CO_2_ immiscible flooding significantly hinder its widespread application. To address these issues, this paper proposes the use of ultrasonic-assisted CO_2_ immiscible flooding to enhance oil recovery (EOR) and analyzes its underlying mechanisms. First, the distribution characteristics of residual oil in CO_2_ immiscible flooding were examined using high pressure mercury injection (HPMI), nuclear magnetic resonance (NMR), and microscopic visualization displacement (MVD) experiments. Next, the changes in oil recovery and residual oil distribution after applying ultrasonic-assisted CO_2_ immiscible flooding were analyzed. Finally, the mechanism of EOR through ultrasonic-assisted CO_2_ immiscible flooding was summarized based on the results of interfacial tension (IFT) tests. The findings indicate that the recovery rate of CO_2_ immiscible flooding without ultrasound is 53.33 %, with residual oil primarily distributed in the form of films and flakes within the pores. The ultrasonic assisted CO_2_ immiscible flooding with frequency of 28 KHz and power of 200 W was provided by ultrasonic horn, and the recovery rate increased by 62.84 % after 60 min. With ultrasonic assistance, the recovery rate increased to 62.84 %, and the maximum radius of residual oil droplets was reduced from 81.8 μm to 36.5 μm. Additionally, the amount of residual oil in the 0.1 μm to 1 μm range slightly decreased, suggesting that ultrasonic assistance mainly improves the recovery of flake residual oil, with a slight improvement in film residual oil recovery as well. Ultrasound can enlarge the pore and throat radius and reduce the interfacial tension in the CO_2_-oil system, allowing more flake residual oil to be displaced. Moreover, the high-frequency vibrations of the ultrasound can reduce the thickness of the oil film, enhancing the recovery of film residual oil. This study supports the further application of ultrasonic-assisted CO_2_ immiscible flooding in both scientific research and engineering practice.

## Highlights

• Ultrasonic assisted CO_2_ immiscible flooding can increase oil recovery by 9.51%.
• Ultrasonic assisted CO_2_ immiscible flooding mainly improves the recovery rate of flake residual oil.
• Ultrasonic assisted CO_2_ EOR main mechanisms: throat size expansion, CO_2_-oil IFT reduction, high frequency vibration.

## 1 Introduction

As the most important greenhouse gas to accelerate climate change and ocean acidification, the Chinese government is seeking to reduce anthropogenic CO_2_ emissions by injecting CO_2_ into oil reservoirs [1], [2]. In addition, the process of CO_2_ flooding has proven to be an effective enhanced oil recovery (EOR) method in unconventional reservoirs [3], [4], [5]. Therefore, CO_2_ flooding is becoming more and more popular in Europe, North America and China [6]. However, it is difficult to achieve CO_2_ miscible flooding due to the pressure of many reservoirs is less than the minimum miscible pressure (MMP) in China [7]. In fact, it has been proven in the laboratory that the recovery of CO_2_ miscible flooding is much greater than that of immiscible flooding [8], [9], [10]. This leads to a series of problems such as low recovery rate, low CO_2_ storage efficiency and poor economic benefit of CO_2_ flooding [11], [12]. Due to the inherent and complex displacement behavior of CO_2_ immiscible flooding, it is of a practical and fundamental importance to examine enhance the recovery of CO_2_ immiscible flooding.

The experimental study shows that with the same displacement pressure, reducing the MMP can improve the recovery of CO_2_ immiscible flooding [13], [14]. The techniques for reducing the minimum miscibility pressure can be classified into two categories: miscible solvents and surfactant methods [15]. The miscible solvents are based on extracting light hydrocarbons from crude oil, increasing the solubility promoting crude oil expansion, reducing oil–water interfacial tension, and other oil displacement mechanisms [16]. The miscible solvent method can be further divided into mono-component and poly-component methods [17]. The mono-component method is to add small molecular hydrocarbons, such as toluene, ethanol and xylene into CO_2_ injection to reduce the MMP and achieve the purpose of improving CO_2_ immiscible flooding recovery [18], [19], [20]. The multi-component method is to inject hydrocarbon solvents such an alcohols, ethers, ketones and esters into the reservoir, and use the interaction between oxygen atoms in hydrocarbon molecules and carbon dioxide to reduce the MMP, thereby improving the recovery of CO_2_ immiscible flooding [21], [22], [23], [24]. Zhao found that with the MMP decreasing from 29.6 MPa to 24.1 MPa by injecting citric acid isopentyl ester which can not only be dissolved in crude oil, but also be dissolved in carbon dioxide, the recovery of CO_2_ immiscible flooding is greatly improved [25], [26], [27]. It can be greatly increased the solubility of CO_2_ in crude oil and reduce the interfacial tension (IFT) of the CO_2_ −crude oil system by injecting surfactants into the reservoir, thus improving the CO_2_ immiscible flooding recovery [28], [29]. Although co-injection of mixed solvents can improve the recovery of CO_2_ immiscible flooding, it is difficult to apply this method in a wide range due to the consideration of safety and economy [30].

In view of the above problems, ultrasonic has been proposed to improve the recovery of CO_2_ immiscible flooding because of its environmental friendliness and remarkable production-increasing effect [31], [32]. In fact, ultrasound has been widely used in the petroleum industry, such as the removal of water from crude oil emulsions [33], [34]. Hossein Hamidi found that improving oil recovery by combining ultrasound application with CO_2_ flooding could be beneficial [35]. However, he only analyzed the effect of ultrasonic-assisted CO_2_ flooding temperature on oil recovery through experimental methods and has not discussed the mechanism of ultrasonic assisted CO_2_ immiscible flooding to enhance oil recovery. Wang confirmed through experiments that under the action of ultrasonic vibration and cavitation, the macromolecules of crude oil to be decomposed into smaller carbon-number molecules, which caused the decrease of viscosity and interfacial tension of CO_2_-crude oil system [5]. The experimental results showed that ultrasound assisted CO_2_ immiscible flooding can increase oil recovery by 11.5 %, but so far, there was no visual experiments directly observing how ultrasonic assisted CO_2_ immiscible flooding improves oil recovery. The purpose of this study is to visually analyze the residual oil distribution phenomenon of ultrasound-assisted CO_2_ immiscible flooding by combining IFT, nuclear magnetic resonance (NMR), microscopic visualization displacement (MVD) and core CO_2_ displacement, and try to explain the mechanism of ultrasound-assisted CO_2_ immiscible flooding to enhance oil recovery. This study has perfected and enriched the theory of ultrasonic assisted CO_2_ flooding and provided the basic theoretical basis for the future wide application of ultrasonic assisted CO_2_ flooding in oil fields.

## 2 Experimental

### 2.1 Materials

**Fig. 1:**
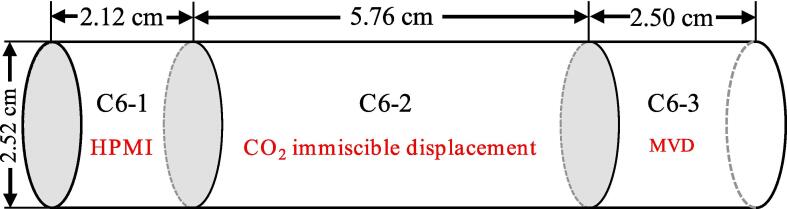
The schematic diagram of core cutting position for different sections.

A core sample with a length of 10.82 cm and a diameter of 2.52 cm was collected in the horizontal direction from Chang-6 sandstone member of the Upper Triassic Yanchang formation in the Ordos Basin, China. To ensure consistent experimental measurements, the core sample was divided into three sections for different experiments (see Fig. 1) by using a wire cutting processing device. From left to right, the first section with a length of 2.12 cm was prepared for the high pressure mercury injection (HPMI) experiment. The second section with a length of 5.76 cm was used for CO_2_ immiscible displacement experiments with the NMR measurements. The third section with a length of 2.50 cm was used for MVD about CO_2_ immiscible flooding experiments. Since some cores are lost during the wire cutting process, the total length of C6-1, C6-2 and C6-3 cores is slightly smaller than the length of the sample before cutting. The organic matter in the core pores was cleaned with a solution composed of alcohol and phenol at a volume ratio of 1:3. Gas permeability, porosity, and other physical properties of different sections are tabulated in Table 1, the uncertainty of the physical properties of different sections is within 2 %, so the visual research of ultrasonic assisted CO_2_ immiscible flooding can be analyzed by combining the experimental results.

**Table 1:** Physical properties of the core samples.

Sample ID	Porosity, %	Permeability, mD	Length, cm	Diameter, cm	Density, g/cm^3^
C6-1	11.34	1.27	2.12	2.52	2.32
C6-2	11.35	1.26	5.76	2.52	2.32
C6-3	11.32	1.27	2.50	2.52	2.31

**Fig. 2:**
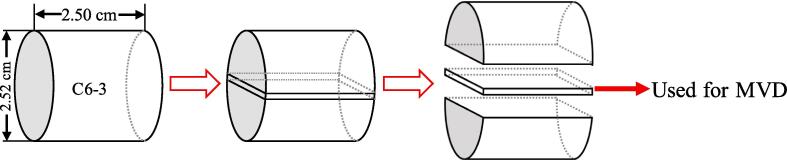
The schematic diagram of coring a slice of rock.

**Fig. 3:**
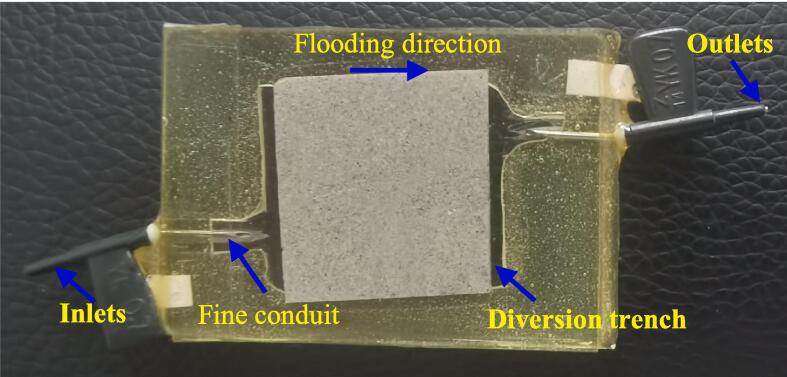
Microscopic displacement model.

The direction of the column core length is parallel to the horizontal direction of the formation. In order to more realistically simulate the displacement in the formation, the C6-3 sample needs to be further processed to meet the requirements of MVD. As shown in Fig. 2, the C6-3 sample was cut from the center of the section to obtain a square sample with a side length of 2.50 cm and a thickness of 0.15 cm. The square sample was then polished into a smooth sheet (surface roughness less than 10 µm) with a thickness of only 0.03 cm. The smooth rock slice was sandwiched between two pieces of plexiglass (Fuyao Glass Industry Group Co., LTD.) and sealed with resin to form a microscopic visual displacement experimental model (Fig. 3). There are inlets and outlets made of fine conduits at both ends of the model to simulate the actual injection and production wells in reservoir development. Also, a diversion trench is also reserved at both ends of the model to ensure that the rock slice is displaced evenly. The maximum pressure and temperature that the sample can withstand are 8.5 MPa and 80 ℃, respectively.

The oil samples were collected from the surface degassing crude oil from the Chang-6 reservoir in the Ordos Basin. The viscosity of the oil is 3.5 mPa·s at 329 K (Reservoir temperature) and atmospheric pressure. The MMP of CO_2_ flooding in Chang 6 reservoir is 14.6 MPa as measured by slim tube experiment. The purity of CO_2_ used in the experiment was 99.9 %. The formation water used in the experiment consists of inorganic salt and deuterium water (D_2_O) with a purity of 99.99 %, depending on the salinity and ion type of the reservoir formation water. The formation water used in the experiment replaced hydrogen atoms with deuterium atoms, shielding the nuclear magnetic resonance signal that can be used to monitor the distribution of oil in the pores of the rock [34].

### 2.2 Experimental setup

**Fig. 4:**
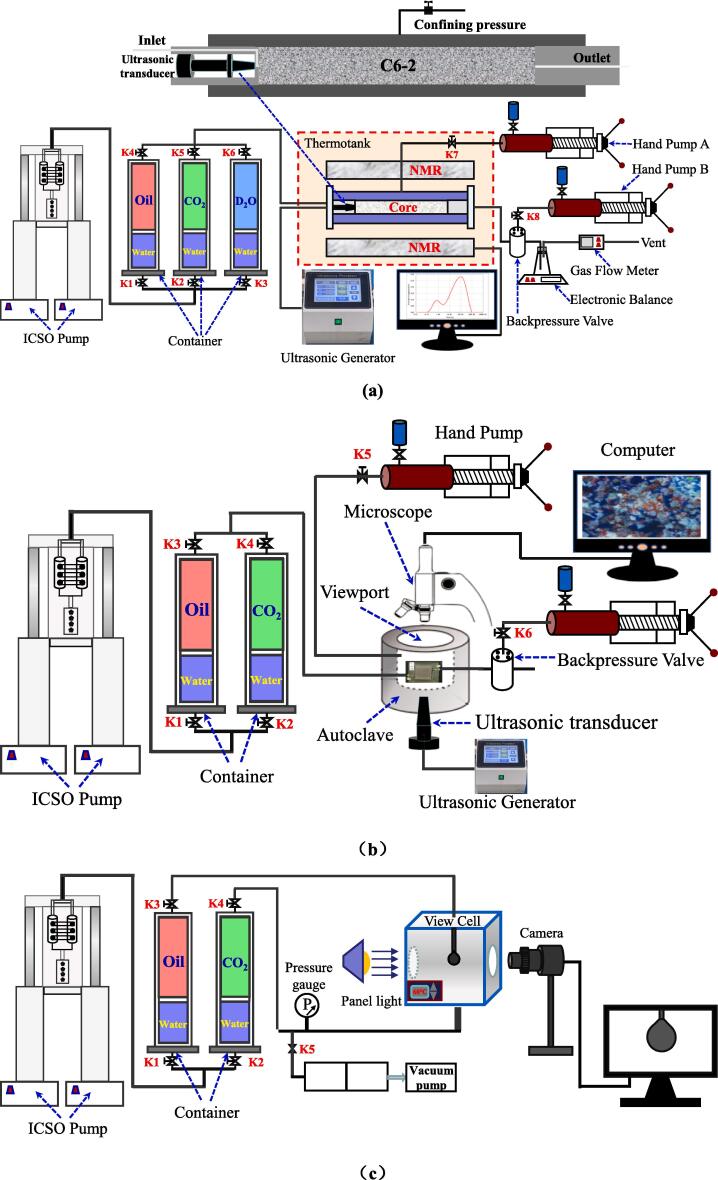
Schematic diagram of (a) integrated NMR and CO_2_ immiscible displacement experiment system; (b) MVD experiment system; and (c) IFT measurement experimental setup of CO_2_-oil system.

As shown in Fig. 4**a**, the experimental setup used for CO_2_ flooding consists of four subsystems: displacement, ultrasonic, NMR instrument and measurement. In the displacement subsystem, oil, CO_2_ and synthetic brine by D_2_O in different containers were injected into a core sample by using a high-pressure syringe pump (100 DX, ISCO Inc.,USA) which can provide the maximum displacement pressure of 70 MPa the flow range is 0.0001 mL/min ∼ 60 mL/min. A core sample was placed in a core holder (P5, 35 MPa, 353 K, Oxford, UK), and the temperature of the core holder was determined using a temperature control unit with an accuracy of 0.2 K. Confining pressure is provided by hand pump (Hai’an, Nantong, China). A backpressure valve with an accuracy of 0.01 MPa is connected to the outlet of the core holder to control the outlet pressure, and the pressure of the back pressure valve is provided by a hand pump. In the ultrasonic subsystem, the ultrasonic generator transmits electrical energy to the ultrasonic transducer attached to the inlet of the core holder and converts it into ultrasonic waves with a frequency of 28 kHz and a power of 200 W. The ultrasonic horn is also equipped with a temperature control system to control the error between the ambient temperature and the experimental temperature of no more than 1℃. In the NMR instrument subsystem, an NMR pulsar (GeoSpec 2/53, Oxford, UK) was used to acquire the transverse relaxation time (T_2_) distributions. The radio frequency is distributed in a range of 1–30 MHz with a control accuracy of 0.1 MHz, while the echo time is set to 0.12 ms, the waiting time for measurements is 1.125 s, and the scan number is 64. In the measuring subsystem, the device consisting of a measuring cup, electronic scale and gas flow meter is used to measure the delivery rate of oil, water and gas with the accuracy is 0.001 ml/min for liquids and 0.01 ml/min for CO_2_ gas.

As shown in Fig. 4**b**, the setup of the experiment for MVD consists of three subsystems: displacement, ultrasound and camera. The pump and container of the MVD experimental setup are the same as those of the CO_2_ immiscible flood experimental setup. The core holder was replaced in the micro subsystem with a hollow cylinder autoclave with a diameter of 12 cm, which has three ports for connecting inlet, outlet and hand pump, each to provide a confining pressure of the micro model. There is a plexiglass viewing window above the autoclave with a maximum pressure of 22 MPa. The ultrasonic transducer is embedded in the bottom of the autoclave, and the power and frequency parameters are the same as the experimental setup for flooding with immiscible CO_2_.

The high-temperature high-pressure interfacial tension setup has been used in the experiments was purchased from S.T. Ltd. France. As shown in Fig. 4**c**, it is mainly consisted of by injection subsystem and camera subsystem. In the injection subsystem, the pump and container of the interfacial tension setup are the same as those of the CO_2_ immiscible flood experimental setup. There is viewport made of sapphire glass on both sides of the high-pressure video box, the inner volume of the high-pressure view cell is 20 cm^3^, and the operating range up to 100 MPa [36]. The camera (Batis 2/40 CF, Zeiss, German) is connected with the computer to transmit the captured image to the computer in time and the image data is automatically analyzed by the software to obtain the interfacial tension of the CO_2_-crude oil system with different pressure.

The instrument model used in HPMI experiment is Autopore IV 9520 automatic mercury porosimeter manufactured by American Instruments. The maximum mercury inlet pressure is 413 MPa, and the measurement accuracy is 0.001 MPa. The mercury injection device is controlled by the computer, which can record the data of mercury inlet volume and pressure during the experiment, and automatically draw the mercury inlet pressure curve.

### 2.3 Experimental procedures

#### 2.3.1 CO_2_ immiscible displacement experiments

After the nuclear magnetic equipment was warmed up for 30 min, C6-2 fully saturated with distilled water by vacuum was placed in the core holder to measure the T_2_ distribution curve reflecting pore size and distribution. Then, C6-2 was placed in the calorstat dried at 373.2 K for 10 h to ensure that the water inside the core was completely evaporated and cooled at room temperature for 10 h before the formation water made of deuterium oxide with purity of 99.99 % was saturated by vacuum. Run the thermotank to keep the experimental temperature stable at 329 K. Sequentially, place C6-2 in the core holder while opening valves 3#, 6#, 7# and 8# in Fig. 4**(a)**, open the high-pressure syringe pump to set the displacement pressure to 1 MPa, increase the outlet pressure to 0.5 MPa with hand pump B, and load the confining pressure to 3 MPa with hand pump A. The formation water in the container continues to enter the core Under the action of the pressure difference between the outlet and the inlet. After 20 min, the displacement pressure is increased to 1.5 MPa while the confining pressure and outlet pressure are also increased by 0.5 MPa to ensure that the confining pressure is always 2 MPa higher than the displacement pressure, and the outlet pressure is always 0.5 MPa lower than the displacement pressure. Increase all pressures by 0.5 MPa every 20 min until the displacement pressure increases to 8 MPa. Close valves 3# and 6# and open valves 1# and 4# to allow crude oil to replace the formation water in the core. Close all valves and pumps and keep the thermostat open to maintain a constant temperature of 329 K until no water is produced at the outlet for 60 min. Sample C6-2 was put into the core holder and aged at a specific temperature of 329 K and pressure of 8 MPa to restore wettability. The T_2_ distributions of accumulated aging time for 168 h was obtained by performing the NMR tests. Since only organic matter contains hydrogen atoms in the pores, the T_2_ spectrum reflects the distribution of crude oil in porous media. Open valves 2#, 5#, 7# and 8#, and run the high-pressure syringe pump to drives CO_2_ in the container to displace the fluid in the core with the pressure of 8 MPa. According to the MMP, the displacement mode was judged to be immiscible. The experimental data were recorded until the experiment was terminated without oil production for 30 min. The pump and all valves were immediately closed, and the T_2_ spectrum reflects the distribution characteristics of residual oil was immediately tested. Then, keep running the high-pressure syringe pump and open alves 2 #, 5 #, 7 # and 8 #, while running the ultrasonic horn and the immiscible CO_2_ displacement was assisted by ultrasound for 60 min.. The temperature and pressure conditions of the experiment are completely the same as those without ultrasound. The T_2_ spectrum reflecting the residual oil distribution characteristics of ultrasonic assisted CO_2_ immiscible flooding was measured immediately after the oil displacement experiment.

#### 2.3.2 MVD

First, the model is vacuumed in a sealed container and saturated with formation water. The model is then placed horizontally in an autoclave filled with water and the inlet and outlet of the model are connected to the interface in the autoclave. Seal the autoclave and wrap a layer of heating cotton around the metal autoclave to maintain the temperature in the autoclave at 329 K (reservoir temperature). Open valve 5# and increase the pressure in the autoclave to 1.5 MPa using hand pump A. Open valve 1#, valve 3# and valve 6# and run the high-pressure syringe pump to inject oil into the model at a pressure of 0.5 MPa. The outlet pressure of the model is increased to 0.28 MPa by hand pump B and backpressure valve. Every 10 min, the displacement pressure, the pressure in the autoclave and the outlet pressure are simultaneously increased by 0.5 MPa until the displacement pressure increases to 8 MPa. If the outlet does not produce water for 15 min, all valves and pumps are closed, and the model is aged for 168 h at experimental temperature and pressure to restore core wettability.

Second, open valves 2#, 4#, 5# and 6# and run a high-pressure pump to force CO_2_ from the container through the line into the model. The experiment was stopped until there was no oil production at the discharge end for 15 min and all valves and pumps were closed. At the same time, the camera subsystem was executed to capture images of all parts of the model, and the photos were enlarged by 1200 times (30 × 40).

Third, repeat the above process while the ultrasonic horn is running to conduct the ultrasound-assisted CO_2_-immiscible flooding visualization experiment. The flooding stopped after 15 min of no oil production and the camera subsystem is reopened to capture the image of the model.

#### 2.3.3 IFT

Before measurement, the high-pressure viewing cell and tubing were completely cleaned with alcohol, then cleaned with distilled water and flushed with dry nitrogen. Make sure there are no organic residues in the cells and tubes. Open valve 5# and the vacuum pump and piston are used to expel air from the high pressure sight cell. Close valve 5, open valve 2 and valve 4 and run the high-pressure syringe pump to fill the cell with CO_2_ and increase the pressure in the cell to 7.75 MPa. Activation wraps the heating sleeve around the surface and increases the temperature inside the cell from room temperature to 329 K. Close valve 2# and valve 4#, after the pressure and temperature stabilize for 20 min, valve 1# and valve 3# are opened closed and the pump pushed 1 μL of crude oil into the cell at a flow rate of 0.001 ml/min, hung it on the capillary tip and then closed the valves 1# and 3#. After the shape of the oil droplets stabilizes, the shape captured by the camera is transferred to the computer so that the software can calculate the interfacial tension. The 50 ml oil in the beaker was treated for 20 min by the probe with ultrasonic wave as shown in Fig. 4**a**. The above experimental process was repeated to measure the interfacial tension between the oil which treated by ultrasonic and the CO_2_ at a pressure of 7.75 MPa.

#### 2.3.4 HPMI

In the course of the HPMI experiment, China's national standard for determining the rock capillary pressure curve (GB/T 29171–2023) was strictly followed.

## 3 Results and discussions

### 3.1 Recovery

In the process of CO_2_ immiscible displacement of core 6–2, NMR was measured a total of four times, and cumulative distribution curves and T_2_ spectral component distribution curves with four different states were obtained. The NMR signals all came from the hydrogen atoms in the water molecules with fully saturated distilled water. Therefore, the number and size of pores are characterized by the T_2_ spectral component distribution curve of the fully saturated distilled water and the cumulative distribution of T_2_ spectrum represents porosity. The second test was performed when both bound water (D_2_O) and saturated oil were present in the pores, since the D_2_O in the core before saturated oil did not contain hydrogen atoms, which represents the T_2_ spectrum from saturated oil to bound water detected by NMR the distribution of oil in the pores and the cumulative pore volume in the pores. The NMR after the end of flooding with immiscible CO_2_ without ultrasound action was measured for the third time. The distribution curve of the T_2_ spectrum components and the cumulative T_2_ spectrum distribution curve represent the size of the residual oil droplets and the total volume of the residual oil, respectively. The NMR after completion of ultrasound-assisted flooding with immiscible CO_2_ was measured for the fourth time.

**Fig. 5:**
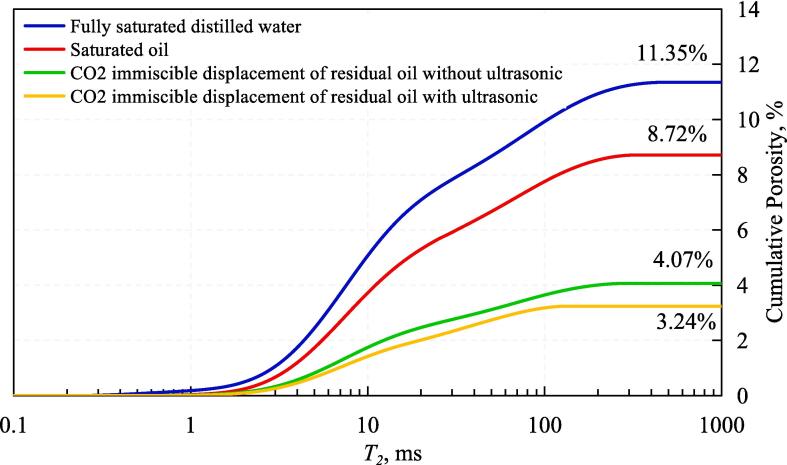
Results of NMR T_2_ spectrum cumulative distribution curve.

The results of the cumulative distribution curve of the T_2_ spectrum of the nuclear magnetic resonance test are shown in Fig. 5. The results of NMR experiment filled with distilled water show that the porosity of C6-2 sample is 11.35 %, and the oil volume accounts for 8.72 % of the apparent volume of the core, which means that the oil saturation is 76.83 %. The residual oil volume of CO_2_ immiscible flooding without ultrasonic is 4.07 % of the apparent core volume, indicating that the residual oil saturation is 35.86 % and the recovery is 53.33 %. The residual oil volume of the ultrasonic assisted CO_2_ immiscible flooding accounted for 3.24 % of the apparent core volume, the calculated residual oil saturation is 28.55 %, and the recovery rate is 62.84 %. Comparing the recovery of CO_2_ immiscible flooding before and after ultrasonic, it is found that the recovery of CO_2_ immiscible flooding is increased by 9.51 % with the action of ultrasonic. It can be seen that ultrasonic assisted CO_2_ immiscible flooding is beneficial to improve recovery.

### 3.2 Characteristics of residual oil distribution

**Fig. 6:**
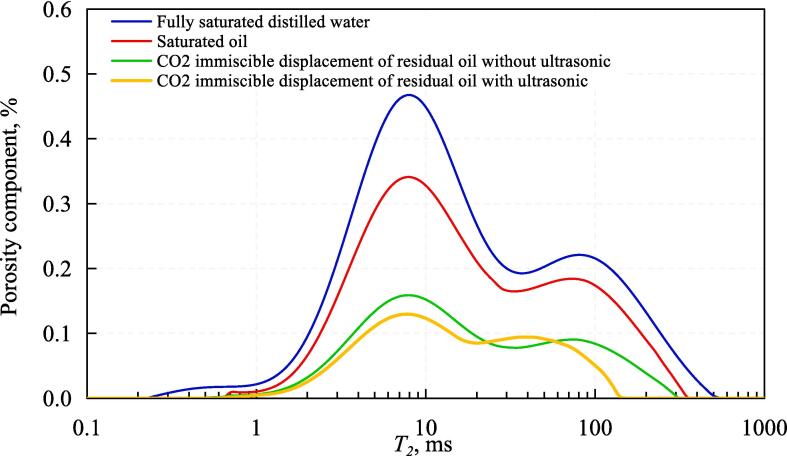
Results of NMR T_2_ spectrum cumulative distribution curve.

The results of T_2_ spectral component distribution curve of nuclear magnetic resonance test are shown in Fig. 6. From Fig. 6, it can be seen that the relaxation time distribution range of fully saturated distilled water is 0.12 ms to 501.2 ms, and the range of saturated oil is reduced to 0.36 to 319.7 ms. The relaxation time of immiscible CO_2_ flooding without ultrasound is further reduced to 0.36 ∼ 281.8 ms, and the distribution range of the relaxation time of ultrasound-assisted immiscible CO_2_ flooding is the smallest at only 0.36 ∼ 141.3 ms. The NMR mechanism shows that when the hydrogen atom in the pore undergoes transverse relaxation motion, the collision between the hydrogen atom and the surface leads to the energy loss of the hydrogen atom. The smaller the pore size, the more frequently collisions occur, the faster the energy loss occurs and the shorter the relaxation time (T_2_). Therefore, it can be assumed that the pore size is proportional to the relaxation time of the hydrogen atom [37], [38], [39], [40]. According to this theory, the distribution curve of NMR-T_2_ spectrum can be converted into an aperture distribution curve by combining with HPMI [41], [42], [43].

**Fig. 7:**
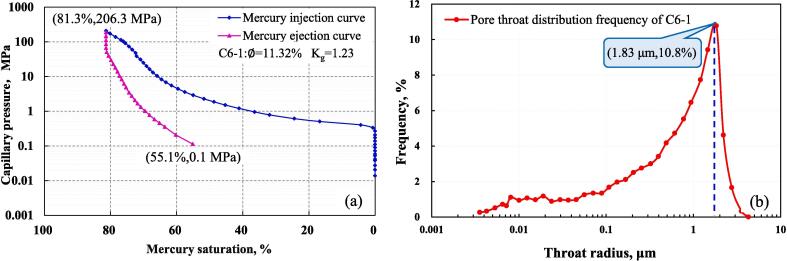
Results of HPMI. (a) capillary pressure curve of C6-1; (b) throat radius distribution curve.

Fig. 7**a** shows the capillary pressure curve of C6-1 obtained by HPMI, the maximum mercury injection pressure is 206.8 MPa, and the corresponding maximum mercury saturation is 81.3 %. When the pressure in the core is reduced to 0.1 MPa, the mercury removal saturation is 55.1 %, and the mercury removal rate is 32.2 %. Fig. 7**b** shows that the C6-1 throat radius distribution curve is unimodal, and the throat with a radius of 1.83 μm accounts for the largest proportion, reaching 10.8 %, and the throat radius distribution in the ranges of 0.004 µm to 3.55 µm.

The relationship between transverse relaxation time and throat radius is [44]:

(1) $$T_{2}=C{\text{r}}_{c}$$

There, *T_2_* is the transverse relaxation time through the pore fluid, ms; *r_c_* is the throat radius, μm; C is the conversion coefficient between T_2_ and r_c_, ms/μm. Therefore, the T_2_ value can be converted into a pore size as long as the C value is determined.

**Fig. 8:**
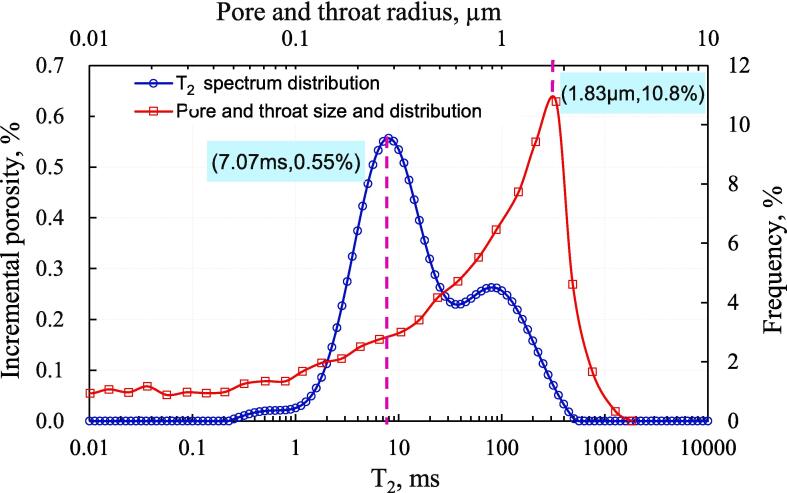
Combination of throat distribution curve and T_2_ spectrum of saturated distilled water.

Fig. 8 shows the superposition of the throat distribution curve and the T_2_ spectrum filled with distilled water. The left peak of NMR T_2_ spectrum represents the distribution of small pores dominated by the throat, and the right peak represents the distribution of medium and large pores. Therefore, the left peak of the NMR T_2_ spectrum should correspond to the peak that also represents the distribution curve of the throat. The T_2_ value corresponding to the left peak position of NMR is 7.07 ms, and the position of the single peak of the throat radius distribution curve is 1.83 μm. According to Eq. 1, the conversion coefficient C can be calculated as 3.86 ms/μm.

**Fig. 9:**
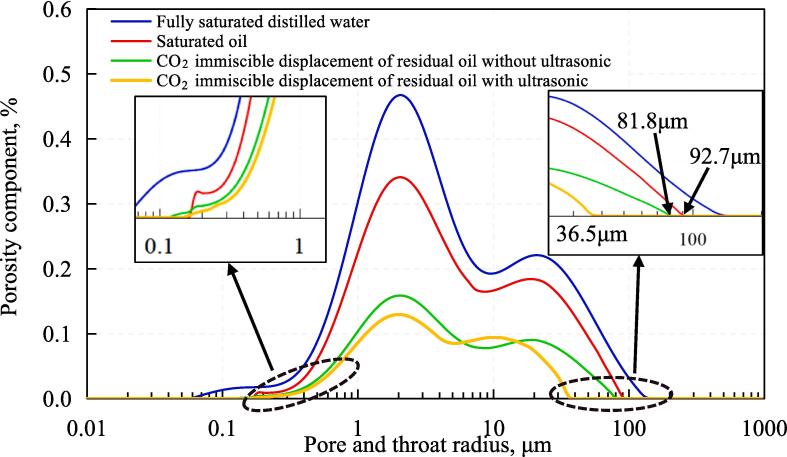
Pore size and frequency distribution obtained by NMR T_2_ spectrum conversion.

**Fig. 10:**
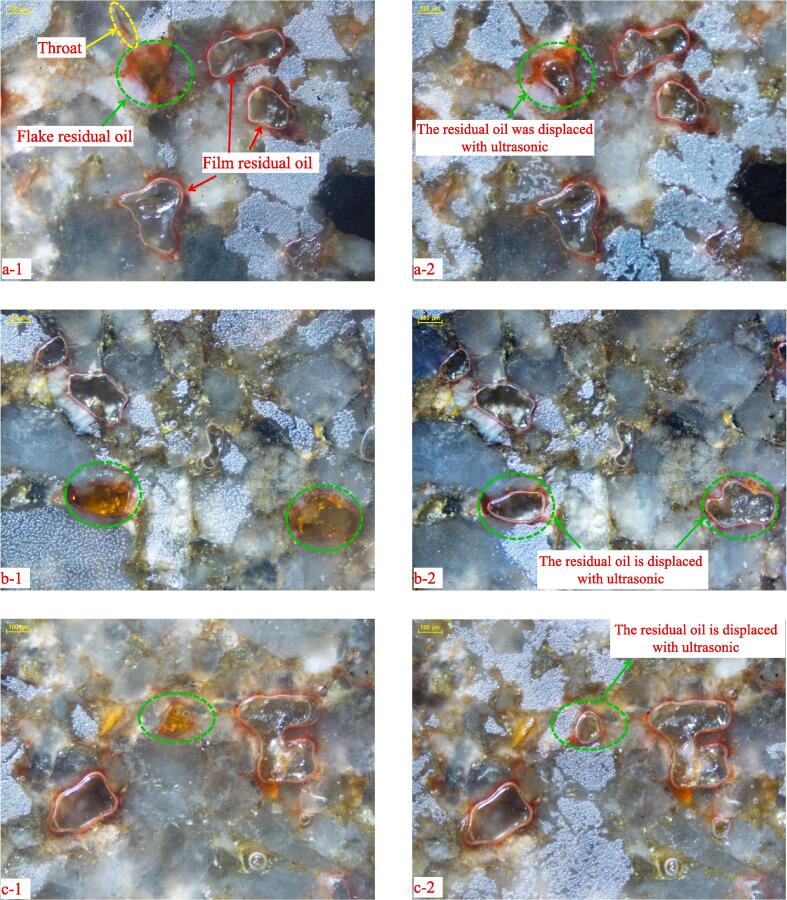
Results of microscopic visualization displacement experiment. (a-1) the first perspective without ultrasonic; (a-2) the first perspective with ultrasonic; (b-1) the second perspective without ultrasonic; (b-2) the second perspective with ultrasonic; (c-1) the third perspective without ultrasonic; (c-2) the third perspective with ultrasonic.

Fig. 9 shows the pore size and frequency distribution curve obtained by combining NMR and HPMI. It can be seen that the original pore size distribution is in the range of 0.065 μm to 129.7 μm, and the oil droplet radius distribution range in the saturated oil state is 0.18 μm to 92.7 μm. The radius distribution of the residual oil with CO_2_-immiscible flooding without ultrasonic action is reduced to 0.13 μm to 81.8 μm. In comparison to the distribution curve in the saturated oil state, it can be seen that the size and quantity of the remaining oil droplets decrease after CO_2_ immiscible flooding without an ultrasonic wave. The maximum size of residual oil droplets is 81.8 μm, which is not much different from the maximum radius of oil droplets in the saturated oil state, indicating that there is CO_2_ flow around or inrush in the process of CO_2_ immiscible flooding without ultrasonic, resulting in the formation of large intact residual oil in part of the pores without being swept by CO_2_. The minimum oil drop radius of 0.13 μm is smaller than that of 0.18 μm in the saturated oil state, indicating that CO_2_ immiscible flooding can only displace part of the crude oil, and part of the crude oil is still in the form of a film on the Rock surface is distributed. These assumptions are confirmed to be correct by microscopic visualization experiments. Fig. 10 shows the results of the microscopic visualization experiment. It can be seen from the figure that the wettability of the rock is oil wet, and the remaining oil can be divided into film and flake forms according to the distribution pattern of the remaining oil.

At the initial stage of CO_2_ immiscible flooding, the water located in the middle of the pores was first displaced by CO_2_ as a non-wetting phase, and then CO_2_ began to displace the oil in the pores under the action of displacement force. The oil adsorbed on the surface of rock pores will form an adsorption layer with a certain thickness, and its viscosity is much larger than that of the free layer due to the oil in the adsorbed layer is mainly composed of macromolecular organic matter. As a result, CO_2_ immiscible flooding cannot completely displace the crude oil in the pores and form film residual oil [45].

**Fig. 11:**
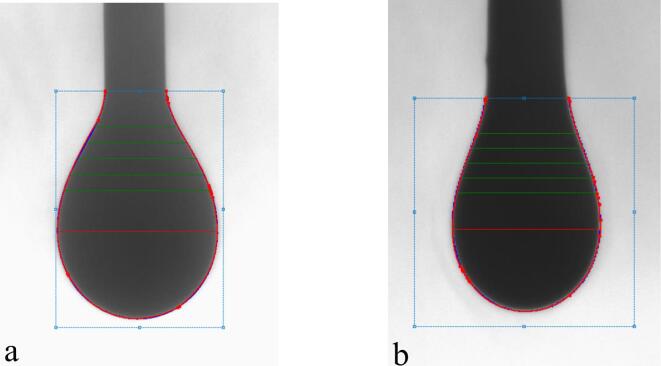
Results of Interfacial tension. (a) Image of the oil that has not been treated by ultrasound, σ_go_ = 11.02 mN/m; (b) Image of oil treated by ultrasound for 20 min, σ_go_ = 8.56 mN/m.

The interfacial tension between CO_2_ and crude oil system without ultrasound is 11.02 mN/m (Fig. 11**a**). As a non-wetting phase, CO_2_ can pass through the neck of the connected pore and enter the pore when the displacement pressure is greater than the capillary pressure. According to Eq. (2), the smaller the neck radius, the greater the capillary pressure [46]. Therefore, as long as the throat radius is small enough, CO_2_ is prevented from entering the pores associated with the throat, resulting in residual oil that cannot be displaced. This is the main reason for the existence of complete flake residual oil in Fig. 10. In general, the oil film thickness is very thin, so the remaining oil of the CO_2_ immiscible flooding is mainly composed of complete flakes. How to displace this type of remaining oil is key to improving recovery from CO_2_ immiscible floods.

(2) $$P_{c}=\frac{2\sigma_{\mathrm{go}}COS\theta }{r}$$

There, P_c_ is capillary pressure, MPa; θ is the contact angle of oil and rock, ^。^; σ_go_ is the interfacial tension between oil and CO_2_, mN/m; r is throat radius, μm.

Compared to the distribution curve of CO_2_ immiscible flooding without ultrasound, it can be seen that the size of the maximum residual oil droplets is further decreased from 81.8 μm to 36.5 μm and the porosity component of the residual oil is reduced in the size range from 18.3 μm to 36.5 μm, meaning the number of remaining oil droplets in this size range is reduced. However, the amount of residual oil does not change significantly in other size ranges, so the ultrasound-assisted CO_2_ immiscible flooding mainly improves the recovery rate of the flake residual oil. In fact, ultrasound can also enhance the recovery rate of film residual oil, but the thickness of film residual oil is only a few microns or even submicrons. From a microscopic perspective, the oil film thinning phenomenon caused by ultrasound is not obvious, which is difficult to directly observe through microscopic visualization experiments. Fortunately, experimental results from nuclear magnetic resonance can support this point of view. For example, the quantity of residual oils is also reduced in the range of 0.1 to 1 μm, indicating that the oil film becomes thinner under the action of ultrasound, resulting in a decrease in the size of the residual oil (Fig. 10).

### 3.3 Mechanism of EOR by ultrasonic-assisted CO_2_ immiscible flooding

**Fig. 12:**
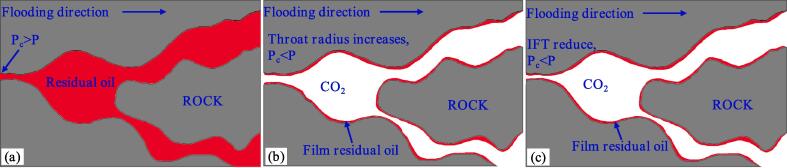
Mechanism diagram of ultrasonic assisted CO_2_ immiscible flooding. (a) Flake residual oil; (b) With the increase of throat radius, the displacement pressure (P) is greater than the capillary force (P_c_), and the crude oil in the pore is displaced by CO_2_ to form film residual oil; (c) As the interfacial tension decreases, the displacement pressure is greater than the capillary force, and the crude oil in the pore is displaced by CO_2_ to form the film residual oil.

Tian found that ultrasonic exposure can increase the size of the pores and neck of the core [47]. According to Eq. (2), Increasing the neck radius leads to a reduction in capillary force under the condition of constant contact angle and constant interfacial tension. Therefore, under the action of ultrasound, CO_2_ penetrates the pores that were originally inaccessible and displaces the crude oil in the pores (Fig. 12**b**). The results of the IFT show that the interfacial tension between the oil which treated by ultrasonic and CO_2_ decreases from 11.02 mN/m to 8.56 mN/m under the condition of reservoir temperature and pressure, and the decrease of interfacial tension also contributes to the decrease of capillary force, resulting in more crude oil being driven out of the pores (Fig. 12**c**). In fact, in the process of ultrasonic assisted CO_2_ immiscible flooding, the increase of pore throat size and the decrease of interfacial tension occur at the same time, which makes a greater contribution to the improvement of oil recovery.

The high-frequency vibration effect of the ultrasonic wave can also cause the residual oil film adsorbed on the rock surface to fall off and become free oil, which is displaced by CO_2_. However, this vibration effect hardly contributes to improved oil recovery. When flooding with immiscible CO_2_, the recovery rate of flaky residual oil in the pores is mainly improved by increasing the size of the pore throat and reducing the interfacial tension.

## 4 Conclusions

Based on this study, the following conclusions can be drawn:

• The recovery of CO_2_ immiscible flooding without ultrasonic action is 53.33 %, and the recovery of ultrasonic assisted CO_2_ immiscible flooding is increased to 62.84 %, so that ultrasonic assisted CO_2_ immiscible flooding is beneficial to improve recovery.
• For oil-wet reservoirs, the range of residual oil size distribution for CO_2_ immiscible flooding is 0.13 μm ∼ 81.8 μm, and the residual oil can be divided into two categories according to the causes and distribution patterns of residual oil. One is the film residual oil adsorbed on the rock surface that cannot be displaced; the other is the flaky residual oil caused by the throat size being too small for CO_2_ to penetrate the pores and displace the crude oil.
• Ultrasound assisted CO_2_ immiscible flooding mainly improved the recovery of flake residue oil, with the maximum cluster size of residual oil droplets decreasing from 81.8 μm to 36.5 μm, and the recovery of film residue oil was also slightly improved by ultrasound.
• Ultrasound can enlarge the pore and throat radius and reduce the interfacial tension of the CO_2_-oil system, allowing more flake residual oil to be displaced. In addition, the high-frequency vibration of the ultrasonic wave can also reduce the oil film thickness and improve the recovery rate of the film residual oil.

## CRediT authorship contribution statement

**Hengli Wang:** Writing – original draft, Investigation, Formal analysis. **Leng Tian:** Validation, Methodology, Investigation, Formal analysis, Conceptualization. **Yanzhong Zhen:** Validation, Methodology, Investigation, Formal analysis. **Yating Li:** Investigation, Formal analysis. **Yi Gao:** Methodology, Investigation. **Gaorun Zhong:** Methodology, Investigation. **Kaiqiang Zhang:** Supervision, Methodology, Conceptualization, Project administration.

## Declaration of competing interest

The authors declare that they have no known competing financial interests or personal relationships that could have appeared to influence the work reported in this paper.
